# Household and context-level determinants of birth registration in Sub-Saharan Africa

**DOI:** 10.1371/journal.pone.0265882

**Published:** 2022-04-08

**Authors:** Anne Lieke Ebbers, Jeroen Smits

**Affiliations:** Global Data Lab, Institute for Management Research, Radboud University, Nijmegen, The Netherlands; University of Salamanca, SPAIN

## Abstract

While according to the United Nations birth registration is a human right, in sub-Saharan Africa (SSA) only half of new-born children currently have their birth registered. To gain insight into the reasons behind this low registration rate, we study the role of determinants at the household, sub-national regional and country level, using self-reported birth registration data on 358,842 children in 40 SSA countries. While most of the variation in reported birth registration is due to factors at the household level, context factors are found to play an important role as well. At the household level, poverty, low education, restricted autonomy of women, and belonging to a traditional religion are associated with lower odds of being registered. Lack of professional care during pregnancy, delivery, and early life also decrease the odds of being registered. Important factors at the context level are the average number of prenatal care visits in the local area, living in an urban area, the kind of birth registration legislation, decentralization of the registration system, fertility rates, and the number of conflicts. To improve registration, the complex dynamics of these factors at the household and context level have to be taken into account.

## 1. Introduction

“Birth registration is a human right, yet less than three quarters of children under 5 years of age worldwide are registered” [1]. Birth registration is included in Sustainable Development Goal 16, in which target 16.9, concerning legal identity, is particularly relevant [2]. Notwithstanding that the problem is broadly recognized, progress in birth registration has been slow and limited [3, 4]. According to UNICEF [5], still a 100 million children will not be registered by 2030, unless progress is accelerated.

The consequences of not having a birth registration are enormous. Research shows that unregistered children have limited access to services, like health care, and cannot be protected by the law [6–8]. There are grave consequences for the child’s future as well, since a birth registration is often needed for acquiring property, employment, social security, and to vote [9–12]. For governments, accurate registration data are an important source of population data, which are essential for the creation and evaluation of services and development strategies [12–14]. It therefore is of the utmost importance to get a comprehensive understanding of the factors by which registration rates are affected.

Until around 1995, most research on birth registration was focused on developed countries [15–17]. Since then, the problem of under-registration in low and middle income countries (LMICs) has become apparent, shifting the focus of research to the causes of non-registration in these countries [18, 19]. However, most of these studies focus on factors at the household level, where decisions regarding registration are generally taken. An important reason for this is that available data are usually derived from household surveys, as only few developing countries have a precise and objective assessment of birth registration coverage [10, 17, 19]. Potentially relevant factors at other levels, like the economic development of the region, a previous colonial regime, or the national legislative framework are often disregarded [7, 11, 20].

However, recent research for India indicates that studying factors at the household and context level simultaneously can significantly improve our understanding of birth registration problems [20]. It therefore is important to also study the role of contextual factors for other countries. In this study, this is done for countries in the SSA region, where the problem of nonregistration is even more pressing [1]. To make this possible, an encompassing framework is developed, including factors at the household, regional and–for the first time also–national level, which are known or expected to influence birth registration outcomes. The predictions of this framework will be tested by applying multilevel logistic regression analyses to a database with information on self-reported birth registration data for 358,842 children living in 809 areas within 40 SSA countries.

In this way, we aim to contribute to the literature in important ways. First, by studying the determinants of birth registration at the household and context level simultaneously, the relative contribution of risk factors at the different levels can be estimated. Second, by using a very large sample of children, more precise estimates of the effects of those risk factors can be obtained. Third, by using data for 40 SSA countries, the role of factors at the national level can be studied better than in earlier research focusing on only one or a few countries.

## 2. Birth registration

Birth registration is defined as: “the continuous, permanent, and universal recording, within the civil registry, of the occurrence and characteristics of births in accordance with the legal requirements of a country” [14]. Birth registration generally entails the following procedure: (1) an official statement of the birth of a child by a spokesman; (2) the registration of the child and birth by some administrative level of the government that coordinates civil registry; and (3) the publication and circulation of a birth certificate [13, 14]. The certificate includes information on the recording, such as the date and place of the birth, the names of the child, parents, and witness of the birth, and some additional relevant information like the nationality [8, 21]. The procedure can be improved by the notification role of hospitals, midwives and local government officials, who can report new births to the administrative level of the government coordinating civil registry [14]. Although healthcare workers can help with registration and notify the government as a control, the decision regarding the legal registration of a child’s birth can only be carried out at the household level by parents or caregivers [8, 14].

When the registration procedure is completed the child is legally existent and has documentation as proof, enabling the protection of other child’s rights as well, such as access to healthcare and education and legal protection from crimes like child labour [7, 8, 21, 22]. Birth registration not only secures rights in childhood, but is also important for securing rights in adulthood, like social security [8, 10–12]. While compliance with these rights cannot be assured, a person faces a higher chance of compliance when having a birth registration [8]. Moreover, identity documents are important for economic advancement since they are often needed for obtaining employment, property, and using public services [9, 10, 23, 24].

Although the benefits of having a birth registration are substantial, only 46% of the children in sub-Saharan Africa is registered [1, 7]. This indicates that the immediate costs of birth registration are experienced as being higher than the future benefits [10, 23, 25]. Nevertheless, both between and within SSA countries, there is substantial variation in registration rates [20, 26], pointing towards the importance of context factors influencing the outcome of the decision [23, 27].

## 3. Determinants of birth registration

The context in which the birth registration decision is made has basically three relevant levels: the household, local/regional and national level [11, 14]. This study will study potential determinants of birth registration on these three levels simultaneously. In the next sections the relevant socioeconomic, demographic and institutional factors at each of these three levels are discussed.

### 3.1 Household level

The birth registration decision is made at the household level, generally by the parents or caregivers of the child involved [14, 19]. Their decision might be influenced by socio-economic and demographic characteristics of the household. One of the most important determinants at this level is household wealth [13, 19]. In most SSA countries, a fee is involved in the birth registration procedure that may prevent poor families from registering their children [3, 13, 14, 19]. Even if parents do not have to pay for registration initially, there might be a fee for late registration [20]. Not only direct costs, but also indirect costs, like travelling and time that cannot be spend on working, may prevent births from being registered [3, 13, 14, 19]. These indirect costs are often higher for poorer families as they tend to live in more disadvantaged neighbourhoods that often are located further away from registration offices and with worse infrastructures [3, 7]. Besides wealth, access to a mobile phone might be beneficial for registration rates as it may supplement traditional media, like radio and TV, as a source of information. Mobile phones may grant access to the internet, which can improve the access to information about birth registration [28]. Moreover, technological advancement may allow for the development of systems in which a birth can be registered by using a mobile phone [22, 29]. In West Africa, these systems are already starting to work and reduce the costs of birth registration [29].

Besides the proper resources, knowledge about the importance and procedures of birth registration is needed [7, 14, 19]. An important factor influencing knowledge is education [19, 30, 31]. The higher the level of education, the higher the chance that parents recognize the value of having a birth registration and know how to register a birth [14, 32]. Indirect effects can also be seen as educated parents often have better educated social networks, which is one of the most common ways to acquire knowledge and advice about birth registration [20, 33].

Demographic factors like the age of the child or family structures may be important too. Identity documents become more important if children grow older, as they may be needed for entering or graduating from school or to access health services [10, 14, 18, 31, 34]. Regarding family structures, missing one or both parents can make birth registration problematic, for example because in some countries legislation requires the father to register a child [13, 17, 19, 35]. A missing mother may lead to disregarding the birth registration, as mothers are primary caregivers regularly [17]. If the mother is present, also her autonomy and bargaining power within the household are important. According to Mohanty & Gebremedhin [20], bargaining power is important, since women with more bargaining power are more likely to spend resources on their children. These authors also consider the mother’s ability to move around as crucial for activities that enhance the welfare of children, such as immunization, health check-ups, and birth registration. Bloom, Wypij, & Das Gupta [36] provide evidence that women with more autonomy seek more antenatal or prenatal care, which leads to better child health outcomes.

The ethnic or religious group to which a household belongs can also influence the birth registration decision [13, 17, 19, 37]. Such groups can have multiple reasons for not registering births, including name giving traditions that may make early birth registration problematic [14, 17, 38] and misgivings about the way birth registrations are handled [20, 26], like with the Rwandan genocide in 1994 or the apartheid regime in South Africa [7, 13, 17, 24]. Language issues may play a role as well, as minorities in the SSA context often have their own language, which can cause registration problems due to language barriers and illiteracy [12, 13, 37].

Besides socio-economic factors, care-related factors are important. According to Fagernäs & Odame [3], “registration offices are often located within health facilities or close to them, which creates a direct connection between health care and registration.” Health care related to pregnancy and early life can increase awareness of the importance of birth registration. Skilled health personnel, seen as a credible source of information, can provide women with information about birth registration, recommend to register the child, help with the paperwork needed and propose a registration office [20, 31]. This is especially important as traditional birth attendants were found to motivate parents less to register births than skilled birth attendants [14, 17, 28, 37]. Moreover, giving birth at home poses more restrictions on birth registration, for example due to travelling costs and not having help with filling in forms. Receiving care or giving birth in an institutional facility is therefore of great importance.

Availability of primary care, provided by health institutions, in the first years of a child’s life is important as well [4, 28]. According to Pelowski et al. [23], “using vaccine delivery (particularly Diphtheria-tetanus-pertussis, DTP) as an occasion to register births may also provide a means of reaching children born outside health facilities.” The same reasoning holds for receiving vitamin A [3, 19]. Health care personnel can discuss the missing birth registration of a child during the administration of vaccination or vitamin supplements [3, 19].

### 3.2 Context factors

Although the birth registration decision is made at the household level, the situation in the regional and national context can influence the outcome of this decision, as the availability and efficacy of services, policies, and infrastructures vary considerably within and across countries [9, 20, 34]. An important characteristic of a region is the degree of urbanization, reflecting the travelling distance to the nearest registry office–which often do not stretch out to underdeveloped and remote areas–and infrastructure within the region, both affecting the costs and the information flow of birth registration [9, 19, 20, 25, 37].

The availability of services, such as health care and education, is also important [20, 39]. Following Corbacho & Osorio Rivas [25], the further the travel distance, the higher the probability of not making use of available services. Related to this, the mother’s inability to move around within the region can also decrease the use of services [20, 36]. This means that less information on and help with birth registration will be obtained and the chance to receive and spread information about registration (spill over effects) is lower too [20, 39, 40]. A better information infrastructure counters these effects.

National context factors can be expected to be more important than local factors, as birth registration legislation is made at the national level. In low income countries, civil registration systems are often underdeveloped due to the lack of economic resources while these resources are needed for the creation and maintenance of good systems [12–14, 17]. This may lead to legislative barriers too: no legislation at all, outdated legislation, or weak enforcement of existing laws as laws are not harmonized [7, 11, 13, 41]. Besides that, the content of the law might be harmful for registration [12, 31, 42]. A registration fee can be detrimental if poverty is a major issue and specified legal time periods can lead to procrastination–when given a lot of time–or can make registration unfeasible for people living under difficult circumstances–when given little time [11, 14, 18, 19, 23].

Another consequence of lack of resources is that the formation of appropriate institutions for birth registration is problematic [7]. Due to the complex and expensive nature of decentralized systems–as different institutions and actors must interact–centralized systems often are preferred, even though these systems are less flexible and less accessible for people living in rural areas [12, 31, 38]. Countries like Bangladesh, Kenya and Tanzania have seen their birth registration rates go up after moving from a centralized to a decentralized system, which has more local registration points and in which less steps are needed to complete the birth registration process [8, 23, 43].

With regard to demographic factors at the national level, both fertility and child mortality may be important. If fertility rates are high, registering every child may be problematic because of the substantial direct and indirect costs of registration [3, 14, 19, 29]. High child mortality rates, on the other hand, may reduce the motivation of parents to bear the registration costs [29, 41].

A final relevant factor might be the history of the country. The colonial era and periods of war and conflict may have long-lasting consequences for institutional arrangements [13, 17, 44]. With regards to colonialism, path dependency may hamper the development of birth registration processes, for example as colonizers introduced birth registration only in specific regions or only for non-Africans [7, 17, 44]. War and conflicts may have devastating effects on existing registration systems as well [13, 29, 41]. Misuse of these systems during (civil) war, may lead to mistrust and breaking down of them [8, 9, 24, 26]. Episodes of genocide, like in Rwanda and the Demographic Republic of the Congo may have particularly long lasting consequences for registration rates [8, 13, 26].

## 4. Data and methods

### 4.1 Data

For this study, combined datasets from the Demographic and Health Surveys (DHS; www.dhsprogram.com) and Multiple Indicator Cluster Surveys (MICS; https://mics.unicef.org/surveys) have been used, which were derived from the Global data Lab (www.globaldatalab.org) [45]. DHS and MICS are large, nationally representative household surveys. For each survey, non-overlapping areas (often enumeration areas) are randomly selected. These areas (called “clusters” henceforth) are usually communities, villages, or city quarters. In the selected clusters, all households are listed and a random sample of 25–30 households is selected for the interviews. To get a maximum discriminatory power, the data of the most recent standard DHS and MICS surveys for SSA countries, that contain the relevant variables, have been pooled.

Our combined dataset contained information on 442,433 children aged 0 to 4 years old who were living in 809 sub-national regions within 40 sub-Saharan African countries. For an overview of the countries and the numbers of subnational regions, see S1 Table. As a consequence of missing cases on the dependent variable, 81,951 observations could not be included in the analysis. Missing cases on independent variables with less than 500 missing were handled by listwise deletion. For this reason 1640 observations were removed. Accordingly, 358,842 observations remained for the analysis. For missing characteristics of parents, birth registration legislation, or conflicts, dummy variable adjustment was used [40, 46]. In accordance with ICF International [47], the standard weights included in the DHS surveys were de-normalized and subsequently normalized according to the national population sizes. In this way a representative sample of the population in the 40 countries was created.

Context factors at the sub-national regional level were constructed by aggregating data from the household surveys to the local area level, using the regional codes present in the datasets [48, 49]. For urban and rural areas of sub-national regions, separate indicators are used. Data on birth registration systems and legislation were derived from UNICEF [50]. Data for national income, rule of law, government effectiveness, the fertility rate, and under-five mortality were derived from the World Bank [51–53]. For South Sudan, no data for 2010 was available for the variable rule of law. Therefore, the year of 2011 was used instead. Information about the history of the country was retrieved from the Uppsala Conflict Data Program [54] Version 19.1 (UCDP) [55, 56]. In the few cases that no information on the colonial period was available at the UCDP, the Encyclopaedia Britannica was used instead [57].

### 4.2 Methods

A three-level multilevel logistic analysis is used to address the clustering of households within sub-national regions and countries [20, 27]. Logistic regression is used as the dependent variable is a binary outcome with value 1 if the child was reported to have its birth registered and value 0 if it was reported to have no registration. The variable is based on the question: “Does (NAME) have a birth certificate? (If no, probe): Has (NAME)’s birth ever been registered with the civil authority?” Children that were reported to have a birth certificate or to be registered obtained a value of 1 and children that were reported to have neither a birth certificate nor a registration obtained a value of 0. The categories ‘don’t know’ and ‘missing’ were marked as missing values.

Following Mohanty & Gebremedhin [20], first two empty models with random effects at the national and local level are estimated. For these models the intraclass correlation will be estimated in order to determine the variation within and across different levels [20, 58]. Following these models, household and context variables are added [27, 40]. For studying the role of the context factors, given the restricted number of regions and countries, an explorative approach is used in which only significant variables are included in the models. At the household level more observations are available, so that statistical significance is less an issue there and effect sizes are more important. We checked for multicollinearity of the interval variables and found all of them to have a VIF value below the critical value of 5. All models were estimated with MLWIN version 3.04.

### 4.3 Independent variables

Household wealth was measured by the International Wealth Index (IWI), which indicates the standard of living of households based on their possession of durable goods, the quality of their housing and access to basic services [59]. Mobile phone ownership is measured by a dummy variable with value 1 if the household owns a mobile phone and value 0 if not. Education of the parents was measured by the years of education they completed.

Regarding demographics, age was measured in years. Missing of one or both parent(s) was measured by two dummy variables, indicating whether (1) or not (0) the father or mother was missing from the household. The position of women in the households was indicated by two variables, a dummy indicating whether (1) or not (0) the mother has given birth before the age of 18 [40] and whether the mother can decide on contraception [20] with three categories indicating whether the decision on contraception is taken by the mother herself (1), the partner (2), or whether it is a joint decision (3).

The variables ethnicity and religion were based on pre-coded questions with the option to add additional categories. Religion consists of 7 categories, namely: (1) Catholic, (2) Protestant, (3) Christian, not specified, (4) Muslim, (5) no religion, (6) other, and (7) Traditional. For ethnicity a three-category variable was constructed indicating whether the ethnicity of the household is a (1) majority group, a (2) normal sized group, or a (3) minority group. These values were given based on the percentage distribution of the existing groups within the country. If a group concerned 0–10% it was seen as a minority, if it concerned 10–30% it was seen as a regular group, and if it concerned 30+% it was seen as a majority.

Prenatal care personnel indicates who has performed the prenatal check: (1) no prenatal check was performed, (2) a traditional health care worker, (3) another person, (4) or skilled health personnel. The place of delivery is indicated by a dummy variable with value 1 when the birth took place at (someone’s) home while value 0 when at an institution. Assistance during delivery was computed to show whether the birth was assisted by: (1) no one, (2) a traditional birth attendant, (3) another person, (4) or skilled health personnel. The variable postnatal check reflects whether (0) or not (1) a postnatal check has been performed within 2 months after the birth. The variable vaccination shows whether children have ever received a vaccination (0) or not (1) while the variable vitamin A reflects whether a child received vitamin A in the last 6 months (0) or not (1).

Context factors at the regional level were aggregated from the household data, following Smits & Huisman [27]. Availability of health facilities was measured by the mean number of prenatal check-up visits of women in the region. The information infrastructure was indicated by the average number of years of education of adult males and the percentage of households with a phone in the region. For the position of women, the mean age at first birth in the region was used. The variable urbanization reflects whether (1) or not (0) the household lives in an urban area according to the definition used in the surveys.

The economic situation of the country was measured by the Gross Domestic Product per capita (in current US dollars). The variable birth registration legislation indicates whether a country has (1) a legislation for birth registration or not (0). In turn, the variable no update in legislation represents whether there has been an update in birth registration legislation over the years (0) or not (1). Time allowed for registration is a dummy variable that indicates that a birth must be registered within a month (1) or not (0). Registration fee is a dummy variable indicating whether (1) or not (0) a fee was involved in birth registration. The organizational structure indicates whether the procedure is decentralized (0) or centralized (1). The level of governance of the country is proxied by the variable rule of law, in which -2.5 is the lowest score and 2.5 the highest [53]. The demographic situation of the country was indicated by the fertility rate and the mortality rate of children under 5 years old. Finally, the history of the country is represented by the number of conflicts between 1990 and the year of the survey, computed by adding non-state conflicts and state conflicts, and whether a country has been colonized (1) or not (0) [55, 60–62].

### 4.4 Descriptive statistics

S2 and S3 Tables show descriptive statistics for the variables used in our analyses. We observe that 52.1% of the children aged 0–4 in the sample did not have their birth registered. The average IWI of the households in this study is 31, showing that asset ownership and quality of housing and services is low in these countries. However, in 67% of the households a phone is present. Of the children, 28% lived in a household where the father is not present and 5% in a household where the mothers is not present. The average years of education is with 4.9 about one year higher for men than for women. Also, 36% of the women, of whom age at first birth is known, have given birth before the age of 18. Of the children with valid information on the care variables, 60% is born in a health institution and 58% is born with help of skilled health personnel while 40% is still born at home and 18% with help of a traditional birth attendant. In other cases, no care is received at all. For 12% of children, the mother received no prenatal care. Regarding the children, 54% has not received a postnatal check-up within 2 months, 30% has not received a vaccination, and 42% has not received a vitamin A supplement in the last 6 months. The households lived predominantly in rural areas (72%). As most countries have birth registration legislation (96%), the content of the legislation is expected to explain most of the variation in registration at the national level.

## 5. Results

**Fig 1:**
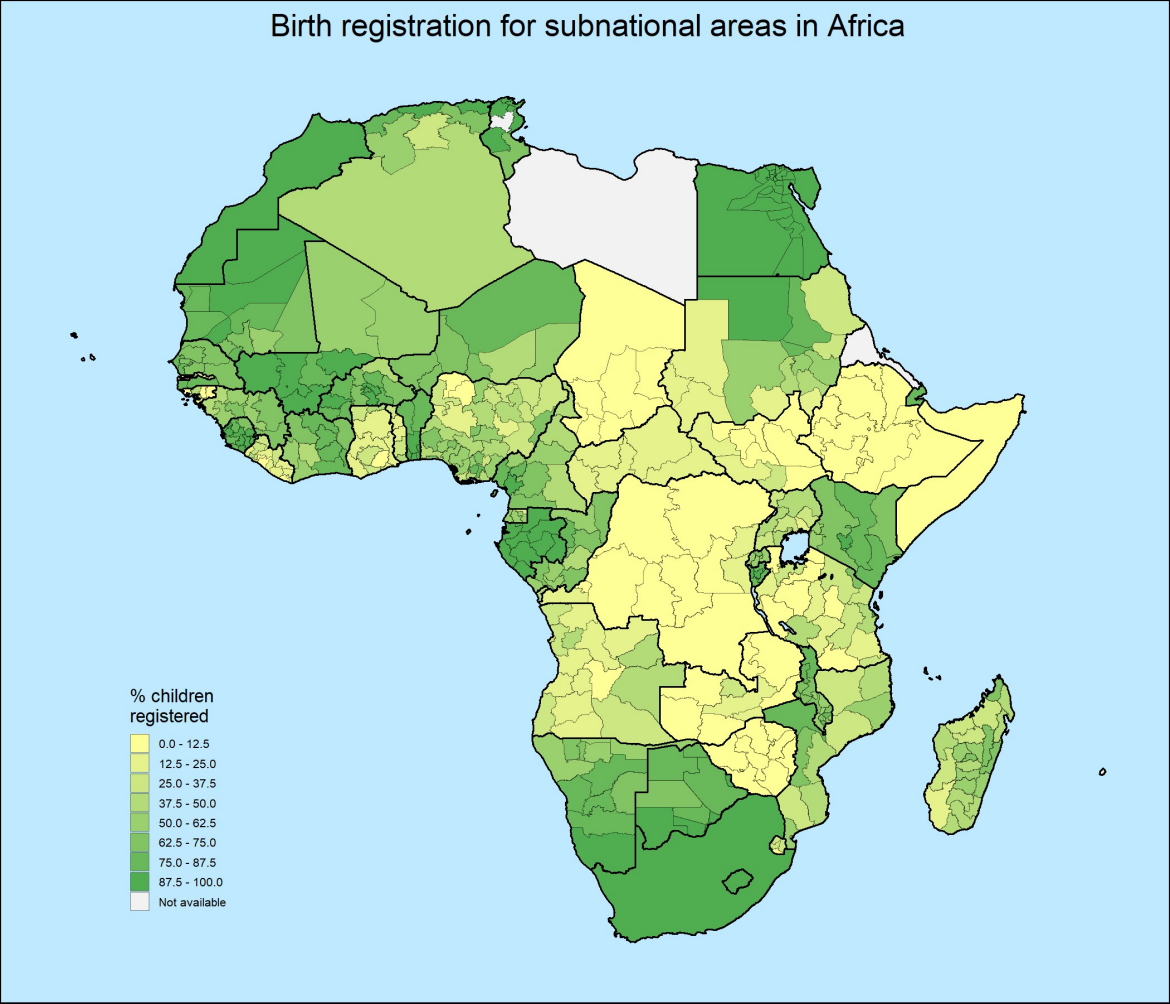
The distribution of countries, years and the number of sub-national regions (provinces).

Fig 1 shows the variation in birth registration percentages across the African continent. Most of the percentages are derived from our database. However, to make the map as complete as possible, we have added subnational data for Egypt (DHS 2014), Tunisia (MICS 2018), Djibouti (MICS 2006) and Algeria (MICS 2019) and national figures for South Africa (2017 from the World Bank) [51], Morocco (2018 from UNICEF) [63] and Somalia (2020 from UNICEF) [63]. For Côte D’Ivoire (2016), Nigeria (2018), Sierra Leone (2019) and Sao Tome en Principe (2019), registration data was derived from a later survey that could not be used in our regression analysis due to data limitations. For Botswana and Equatorial Guinea, sub-national variation from survey data from 2000 was applied to national data from UNICEF [63] to obtain estimates for 2017 and 2011 respectively.

In Fig 1 we see huge differences in registration rates across the continent. For instance South Africa, Botswana, Egypt, Morocco and Kenya have relatively high registration rates, while Chad, Ethiopia, Democratic Republic of the Congo and Zimbabwe have very low rates. Those countries with low rates also show little subnational variation, while within-country variation is clearly present in countries like Sudan, Nigeria, Madagascar and Angola. The situation seems to be most problematic in the Landlocked countries of Central Africa and in the Horn of Africa. Central and East Africa.

Looking at the map as a whole, it becomes clear that the variation among countries is larger than the variation within countries. This observation is confirmed if we look at the intraclass correlation coefficients (ICC) of intercept only models [58]. Of the total variation in birth registration in SSA, 61% is due to differences at the household level and 29% due to differences between countries, whereas only 10% of the variation is due to differences between sub-national regions.

### 5.1 Regression analysis

Table 1 presents the results of the multilevel logistic regression analysis. The table shows that most of the household level factors have the expected effect: having more wealth, a higher age, and more years of education as a parent all significantly increase the odds of having a birth registration. The coefficients of the variables phone, father missing, and mother missing show no significant effects. Also the variables regarding decision making about contraceptive use are not significant. Nevertheless, the position of women in the household seems important for birth registration, as children of a mother who has had her first birth under the age of 18 have significantly lower odds of being registered. Whether you belong to a major, regular, or minor ethnic group within the country is not important. However, belonging to a religious group seems to play a role. Children that belong to Catholic, Protestant, Christian, or Islamic families have higher odds of having a birth registration than children from families with a traditional religion. Children with a Catholic background have the highest odds of being registered.

**Table 1:** Logistic fixed effects models with random intercepts ^a^.

	Odds ratio		Log odds		Standard error	95% CI lower		95% CI upper	
Fixed Intercept	0.074*		-2.604		1.199	-4.954		-0.254	
Province level intercept	1.467**		0.383		0.095	0.198		0.569	
National level intercept	2.601**		0.956		0.221	0.523		1.389	
**Household level**									
International Wealth Index	1.013**		0.013		0.002	0.010		0.016	
Household has phone	1.053		0.052		0.034	-0.015		0.119	
Child’s age	1.053*		0.052		0.021	0.011		0.094	
Years of education father	1.021**		0.021		0.004	0.013		0.030	
Years of education mother	1.028**		0.028		0.005	0.017		0.038	
Father not present	1.055		0.054		0.064	-0.072		0.179	
Mother not present	1.030		0.030		0.077	-0.121		0.180	
Age at first birth age 18 (+)	Reference		Reference		Reference	Reference		Reference	
Age at first birth before age 18	0.940**		-0.062		0.015	-0.092		-0.032	
Partner usually decides on contraception	Reference		Reference		Reference	Reference		Reference	
Mother usually decides on contraception	0.951		-0.050		0.056	-0.160		0.059	
Joint decision mother and partner	0.979		-0.021		0.057	-0.132		0.090	
Ethnicity majority group 30(+)%	Reference		Reference		Reference	Reference		Reference	
Ethnicity regular group 10–30%	0.999		-0.001		0.069	-0.137		0.134	
Ethnicity minority group 0–10%	0.970		-0.030		0.058	-0.143		0.084	
Religion Traditional	Reference		Reference		Reference	Reference		Reference	
No religion	1.028		0.028		0.067	-0.104		0.160	
Religion Catholic	1.224**		0.202		0.044	0.116		0.289	
Religion Protestant	1.096*		0.092		0.046	0.003		0.181	
Religion Christian, not specified	1.107**		0.102		0.036	0.031		0.174	
Religion Muslim	1.151*		0.141		0.067	0.010		0.272	
Religion Other	1.075		0.072		0.057	-0.040		0.184	
Prenatal care traditional health care worker	Reference		Reference		Reference	Reference		Reference	
Prenatal care skilled personnel	0.983		-0.017		0.056	-0.127		0.092	
No prenatal care	0.698**		-0.360		0.095	-0.547		-0.174	
Prenatal care other personnel	0.931		-0.072		0.051	-0.172		0.027	
Delivery at health institution	Reference		Reference		Reference	Reference		Reference	
Delivery at home	0.823**		-0.195		0.059	-0.310		-0.080	
Traditional birth attendant	Reference		Reference		Reference	Reference		Reference	
Skilled birth attendant	1.162*		0.150		0.067	0.020		0.281	
No delivery assistance	0.953		-0.048		0.077	-0.198		0.102	
Other birth attendant	0.980		-0.020		0.042	-0.101		0.062	
Postnatal check within 2 months	Reference		Reference		Reference	Reference		Reference	
No postnatal check within 2 months	0.927*		-0.076		0.036	-0.146		-0.005	
Had vaccination	Reference		Reference		Reference	Reference		Reference	
Never had vaccination	0.751**		-0.286		0.072	-0.427		-0.145	
Received vitamin A in last 6 months	Reference		Reference		Reference	Reference		Reference	
Not received vitamin A in last 6 months	0.869**		-0.140		0.047	-0.231		-0.049	
**Local context**									
Average number of visits antenatal care region	1.097**		0.093		0.020	0.054		0.132	
Urban or rural area	1.096*		0.092		0.046	0.002		0.181	
**National context**									
GDP per capita	1.0002**		0.0002		0.00007	0.00009		0.0004	
Birth registration centralized	0.283**		-1.263		0.319	-1.889		-0.638	
Fee for birth registration	0.334**		-1.096		0.303	-1.689		-0.503	
Fertility rate, total (births per woman)	1.621*		0.483		0.234	0.025		0.941	
Number of conflicts	0.976*		-0.024		0.010	-0.044		-0.004	
Valid N: 358,842									

* P<0.05

** P<0.01.

^a^ Dummy variable adjustment indicators are not presented (see S4 Table).

Among the care variables at the household level, we see that children born at a health institution, with help of skilled health personnel, who were checked within 2 months after birth, who have a vaccination, and who have received vitamin A in the last 6 months all have significantly higher odds of being registered. Receiving prenatal care is also important, but whether it is given by a professional or a traditional health care worker does not make much difference.

Regarding context factors at the sub-national regional level, both the average number of prenatal care visits in the region and urbanization show significant effects. As expected, registration rates are higher among children living in urban areas and in areas in which the average number of prenatal care visits is higher. If the average number of prenatal care visits increases by 1, or one lives in an urban area, the odds of having a birth registration increase by about 10%. No significant effects are found for the age at first birth of women in the region, the educational level and the percentage of households with a phone in the region.

With respect to the variables at the national level, GDP per capita shows a significantly positive relationship with birth registration. Moreover, the organization of the birth registration system is important. In countries with a centralized system, the odds of having a birth registered are 72% lower, and in countries where a fee has to be paid they are 67% lower. Next to the content of birth registration legislation, the fertility rate in the country and the number of conflicts experienced in recent decades are significantly associated with registration. Birth registration rates are higher in countries with higher fertility rates and in countries that experienced less conflicts.

## 6. Conclusion & discussion

Lack of birth registration is a major problem in many LMICs [1]. Most research on the causes of this phenomenon focus on factors at the household level, although factors at higher levels are arguably important as well [7, 11, 20]. This paper contributes to the literature by investigating the determinants of birth registration at the household, sub-national regional and national level simultaneously, on the basis of data for 358,842 children aged 0–4 in 40 SSA countries. Our multilevel logistic regression analyses revealed that most (61%) of the variation in birth registration rates can be explained by factors at the household level, but that also a substantial part of 29% is related to factors at the country level. Sub-national regional factors play a smaller role, explaining only 10% of total variation in birth registration.

At the household level, both socio-economic, demographic and care-related factors are important. Children from wealthier households, older children, children with more highly educated parents, children with a mother who was over age 18 when her first child was born, and children from families with a non-traditional religion have significantly higher odds of being registered than other children. Although the variable phone is not significant in the current model, its role may become more important in the coming years given the increasing penetration of mobile phones with access to internet.

Regarding the role of (reproductive) health care related factors, most findings are in line with the expectations. Children from households that make less use of health facilities and of skilled health personnel have lower odds of being registered. Not receiving prenatal care during pregnancy is especially problematic. In line with these findings, the average number of prenatal care visits at the sub-national regional level is associated with more birth registration as well. Other relevant context factors are urbanization, GDP per capita, centralization of the birth registration system, a fee for birth registration, the fertility rate, and the number of conflicts. Urbanization, GDP per capita, and the fertility rate all increase the odds of having a birth registration, while a centralized birth registration system, a fee for birth registration, and the number of conflicts all decrease the odds of having a birth registration.

Given the large number of involved factors at different levels of analysis, we can conclude that the birth registration problem is complex and highly context specific. Underestimation of this complexity and the role of the context might be important reasons for the limited and slow progress made in achieving universal birth registration [3, 4, 7, 64]. Our findings are, therefore, highly relevant for policy makers. Previous research has recommended to focus on household wealth, education, and access to governmental services such as health care [8, 19, 28]. The results of our analyses indeed show these factors to be important. However, we also found that children of mothers with a weak bargaining position within the household, children from families that belong to a traditional religion, and children that do not have access to health facilities are at a disadvantage. These families may be hard to reach, but reaching them seems essential for increasing registration rates. This could for example be done by an active outreach program that sends mobile units to hard-to-reach areas and by working together with local agents and organizations, such as NGOs, religious organizations and community leaders [19, 65, 66].

Factors at the household level are only part of the story, and are probably the most difficult to change. Policies should therefore also focus on context factors, like those related to the local health care system and the national birth registration system and regulations. According to Muñoz et al. [67], to improve the Civil Registration and Vital Statistics (CRVS) system one should not only focus on technological adjustments, but also on the system itself in order to be efficient and effective. The Ten CRVS Process Milestones mentioned in their paper make clear that active notification by hospitals, as well as grouping activities like registration and certification together, may be helpful for making the system more efficient and effective. The results in this paper show that also decentralization of the system and removing fees in the birth registration process may be important for improving the registration rates.

A limitation of our study is that the birth registration question in the DHS and MICS surveys does not ask the parents for evidence of registration, which means that the answers may suffer from misreporting due to desirability bias and misremembering [4, 14, 31]. According to Adair & Lopez [68], additional problems can be the overestimation of self-reported birth registration rates due to fear for penalties, confusion about birth registration, and not taking deceased children into account. Given these problems, we cannot confidently conclude that the registration rates presented here do not contain any of these potential measurement errors. Our overall SSA registration rate of 48% is rather close to the 46% reported by UN (2019) for the period 2010–2018, however the data on which the UN figures are based are similar to the data used in the current study.

In sum, our simultaneous analysis of the major risk factors at the household, sub-national regional and national level and our finding that 29% of the variation in birth registration is due to factors at the context level constitute major steps forward in the birth registration literature. In particularly the identified associations between context factors and birth registration may have important implications for policy-makers.

## Supporting information

S1 Table The distribution of countries, years and the number of sub-national regions (provinces). (DOCX)

S2 Table Descriptive statistics of household factors. (DOCX)

S3 Table Descriptive statistics of context factors. (DOCX)

S4 Table Full results of multilevel logistic analysis. (DOCX)
