# Supplementary material for: Household and context-level determinants of birth registration in Sub-Saharan Africa
Source: PLoS One. 2022 Apr 8;17(4):e0265882. doi: 10.1371/journal.pone.0265882 (PMC8993011; doi:10.1371/journal.pone.0265882)
Supplement: S1 Table — (DOCX) [file pone.0265882.s001.docx]

| **Country** | **Year** | **Nr. of regions** | **Source** |
| --- | --- | --- | --- |
| Angola | 2016 | 36 | DHS |
| Burundi | 2017 | 10 | DHS |
| Benin | 2018 | 12 | DHS |
| Burkina Faso | 2010 | 26 | DHS |
| Central African Republic | 2019 | 11 | MICS |
| Cote d’Ivoire | 2011 | 20 | DHS |
| Cameroon | 2018 | 19 | DHS |
| Congo Democratic Republic | 2018 | 21 | MICS |
| Congo | 2015 | 15 | MICS |
| Comoros | 2012 | 6 | DHS |
| Ethiopia | 2016 | 21 | DHS |
| Gabon | 2012 | 19 | DHS |
| Ghana | 2017 | 20 | MICS |
| Guinea | 2018 | 15 | DHS |
| Gambia | 2018 | 14 | MICS |
| Guinea-Bissau | 2019 | 17 | MICS |
| Kenya | 2014 | 15 | DHS |
| Liberia | 2013 | 30 | DHS |
| Lesotho | 2018 | 20 | MICS |
| Madagascar | 2018 | 12 | MICS |
| Mali | 2018 | 15 | DHS |
| Mozambique | 2011 | 21 | DHS |
| Mauritania | 2015 | 24 | MICS |
| Malawi | 2016 | 26 | DHS |
| Namibia | 2013 | 26 | DHS |
| Niger | 2012 | 14 | DHS |
| Nigeria | 2013 | 73 | DHS |
| Rwanda | 2015 | 10 | DHS |
| Sudan | 2014 | 30 | MICS |
| Senegal | 2019 | 20 | DHS |
| Sierra Leone | 2013 | 27 | DHS |
| South Sudan | 2010 | 20 | MICS |
| Sao Tome en Principe | 2009 | 8 | DHS |
| Swaziland | 2014 | 7 | MICS |
| Chad | 2019 | 15 | MICS |
| Togo | 2017 | 11 | MICS |
| Tanzania | 2015 | 49 | DHS |
| Uganda | 2016 | 17 | DHS |
| Zambia | 2018 | 18 | DHS |
| Zimbabwe | 2019 | 19 | MICS |
| **Only used for map in figure 1** |  |  |  |
| Algeria | 2019 | 7 | MICS |
| Botswana | 2017 | 10 | MICS |
| Cote d’Ivoire | 2016 | 10 | MICS |
| Djibouti | 2006 | 2 | MICS |
| Egypt | 2014 | 22 | DHS |
| Equatorial Guinea | 2011 | 5 | MICS & UNICEF |
| Morocco | 2018 | - | UNICEF |
| Nigeria | 2018 | 37 | DHS |
| Sierra Leone | 2019 | 14 | DHS |
| Sao Tome en Principe | 2019 | 4 | MICS |
| Somalia | 2020 | - | UNICEF |
| South Africa | 2017 | - | WORLD BANK |
| Tunisia | 2018 | 6 | MICS |

Note: for the map no distinction was made between rural and urban areas while it was for the regression analysis
