# Supplementary material for: Household and context-level determinants of birth registration in Sub-Saharan Africa
Source: PLoS One. 2022 Apr 8;17(4):e0265882. doi: 10.1371/journal.pone.0265882 (PMC8993011; doi:10.1371/journal.pone.0265882)
Supplement: S2 Table — (DOCX) [file pone.0265882.s002.docx]

| **Variables** | **Mean** | **St. Deviation** | **Minimum** | **Maximum** |
| --- | --- | --- | --- | --- |
| Birth registration | 0.479 | 0.500 | 0.00 | 1.00 |
| International Wealth Index (household wealth) | 31.03 | 21.25 | 0.00 | 100.00 |
| Household has phone | 0.666 | 0.471 | 0.00 | 1.00 |
| Child’s age | 1.980 | 1.424 | 0.00 | 4.00 |
| Years of education father | 4.881 | 4.062 | 0.00 | 17.00 |
| Years of education father missing | 0.288 | 0.453 | 0.00 | 1.00 |
| Years of education mother | 3.838 | 4.125 | 0.00 | 17.00 |
| Years of education mother missing | 0.059 | 0.235 | 0.00 | 1.00 |
| Father not present | 0.280 | 0.449 | 0.00 | 1.00 |
| Mother not present | 0.053 | 0.225 | 0.00 | 1.00 |
| Age at first birth before age 18 | 0.317 | 0.465 | 0.00 | 1.00 |
| Age at first birth 18+ | 0.568 | 0.495 | 0.00 | 1.00 |
| Age at first birth missing | 0.115 | 0.320 | 0.00 | 1.00 |
| Mother usually decides on contraception | 0.037 | 0.190 | 0.00 | 1.00 |
| Partner usually decides on contraception | 0.017 | 0.129 | 0.00 | 1.00 |
| Joint decision mother and partner | 0.103 | 0.304 | 0.00 | 1.00 |
| Information contraception missing | 0.843 | 0.364 | 0.00 | 1.00 |
| Ethnicity majority group 30(+)% | 0.267 | 0.442 | 0.00 | 1.00 |
| Ethnicity regular group 10-30% | 0.240 | 0.427 | 0.00 | 1.00 |
| Ethnicity minority group 0-10% | 0.222 | 0.416 | 0.00 | 1.00 |
| Ethnicity missing | 0.272 | 0.445 | 0.00 | 1.00 |
| No religion | 0.031 | 0.173 | 0.00 | 1.00 |
| Religion Catholic | 0.138 | 0.345 | 0.00 | 1.00 |
| Religion Protestant | 0.150 | 0.357 | 0.00 | 1.00 |
| Religion Christian, not specified | 0.135 | 0.341 | 0.00 | 1.00 |
| Religion Muslim | 0.251 | 0.433 | 0.00 | 1.00 |
| Religion Traditional | 0.017 | 0.128 | 0.00 | 1.00 |
| Religion Other | 0.010 | 0.099 | 0.00 | 1.00 |
| Religion missing | 0.270 | 0.442 | 0.00 | 1.00 |
| Prenatal care skilled personnel | 0.460 | 0.498 | 0.00 | 1.00 |
| No prenatal care | 0.069 | 0.254 | 0.00 | 1.00 |
| Prenatal care traditional health care worker | 0.027 | 0.162 | 0.00 | 1.00 |
| Prenatal care other personnel | 0.017 | 0.129 | 0.00 | 1.00 |
| Prenatal care personnel missing | 0.425 | 0.660 | 0.00 | 1.00 |
| Delivery at health institution | 0.482 | 0.500 | 0.00 | 1.00 |
| Delivery at home | 0.320 | 0.467 | 0.00 | 1.00 |
| Delivery information missing | 0.198 | 0.399 | 0.00 | 1.00 |
| Skilled birth attendant | 0.467 | 0.499 | 0.00 | 1.00 |
| No delivery assistance | 0.037 | 0.188 | 0.00 | 1.00 |
| Traditional birth attendant | 0.147 | 0.354 | 0.00 | 1.00 |
| Other attendant | 0.153 | 0.360 | 0.00 | 1.00 |
| Information birth attendant missing | 0.199 | 0.399 | 0.00 | 1.00 |
| Postnatal check within 2 months | 0.210 | 0.407 | 0.00 | 1.00 |
| No postnatal check within 2 months | 0.245 | 0.430 | 0.00 | 1.00 |
| Postnatal check information missing | 0.545 | 0.498 | 0.00 | 1.00 |
| Received vaccination | 0.192 | 0.395 | 0.00 | 1.00 |
| Never had vaccination | 0.082 | 0.274 | 0.00 | 1.00 |
| Vaccination information missing | 0.724 | 0.447 | 0.00 | 1.00 |
| Received vitamin A in last 6 months | 0.367 | 0.482 | 0.00 | 1.00 |
| Not received vitamin A in last 6 months | 0.269 | 0.443 | 0.00 | 1.00 |
| Vitamin A information missing | 0.364 | 0.481 | 0.00 | 1.00 |
| Valid N: 358,842 |  |  |  |  |
