# Supplementary material for: Household and context-level determinants of birth registration in Sub-Saharan Africa
Source: PLoS One. 2022 Apr 8;17(4):e0265882. doi: 10.1371/journal.pone.0265882 (PMC8993011; doi:10.1371/journal.pone.0265882)
Supplement: S3 Table — (DOCX) [file pone.0265882.s003.docx]

| **Variables** | **Mean** | **St. Deviation** | **Minimum** | **Maximum** |
| --- | --- | --- | --- | --- |
| **Local context** |  |  |  |  |
| Average years education fathers in region | 4.938 | 2.091 | 0.522 | 12.82 |
| Average number of visits antenatal care region | 4.107 | 1.025 | 2.084 | 11.65 |
| Percentage of households with a phone region | 0.676 | 0.253 | 0 | 1 |
| Mean age at first birth in the region | 19.22 | 1.053 | 17 | 25.39 |
| Urban area | 0.279 | 0.448 | 0 | 1 |
| **National context** |  |  |  |  |
| GDP per capita | 1397.56 | 1480.31 | 293.00 | 9813.51 |
| Birth registration legislation | 0.963 | 0.190 | 0 | 1 |
| No legislation (dummy variable adjustment) | 0.038 | 0.190 | 0 | 1 |
| Birth registration legislation not updated | 0.500 | 0.500 | 0 | 1 |
| Birth registration legislation updated | 0.462 | 0.499 | 0 | 1 |
| Birth registration centralized | 0.509 | 0.500 | 0 | 1 |
| Birth registration decentralized | 0.454 | 0.498 | 0 | 1 |
| Time allowed for registration – within 1 month | 0.448 | 0.497 | 0 | 1 |
| Time allowed for registration – 1+ months | 0.515 | 0.500 | 0 | 1 |
| Fee for birth registration | 0.244 | 0.429 | 0 | 1 |
| No fee for birth registration | 0.740 | 0.439 | 0 | 1 |
| Information on fee missing | 0.016 | 0.126 | 0 | 1 |
| Rule of law | -0.790 | 0.480 | -1.784 | 0.267 |
| Fertility rate, total (births per woman) | 5.050 | 0.794 | 3.097 | 7.376 |
| Mortality rate, under-5 (per 1,000 live births) | 82.733 | 28.152 | 41.3 | 136.7 |
| Number of conflicts | 14.758 | 21.115 | 0 | 68 |
| Ever been colonized | 0.949 | 0.221 | 0 | 1 |
| Valid N: 358,842 |  |  |  |  |
