# Supplementary material for: Household and context-level determinants of birth registration in Sub-Saharan Africa
Source: PLoS One. 2022 Apr 8;17(4):e0265882. doi: 10.1371/journal.pone.0265882 (PMC8993011; doi:10.1371/journal.pone.0265882)
Supplement: S4 Table — (DOCX) [file pone.0265882.s004.docx]

|  | **Coef.** | **Std. Err.** | **z** | **P>z** | **95% Conf.** | **Interval** |
| --- | --- | --- | --- | --- | --- | --- |
| Fixed intercept | -2.6044 | 1.1990 | -2.1700 | 0.0300 | -4.9544 | -0.2544 |
| IWI | 0.0129 | 0.0017 | 7.5500 | 0.0000 | 0.0096 | 0.0163 |
| Phone | 0.0518 | 0.0340 | 1.5200 | 0.1280 | -0.0149 | 0.1185 |
| Age | 0.0524 | 0.0212 | 2.4700 | 0.0140 | 0.0108 | 0.0940 |
| Years education father | 0.0212 | 0.0043 | 4.8800 | 0.0000 | 0.0127 | 0.0298 |
| Education father missing | -0.0973 | 0.0567 | -1.7200 | 0.0860 | -0.2084 | 0.0137 |
| Years education mother | 0.0275 | 0.0054 | 5.0800 | 0.0000 | 0.0169 | 0.0382 |
| Education mother missing | 0.0491 | 0.0621 | 0.7900 | 0.4290 | -0.0727 | 0.1708 |
| Father missing | 0.0536 | 0.0638 | 0.8400 | 0.4010 | -0.0715 | 0.1787 |
| Mother missing | 0.0298 | 0.0768 | 0.3900 | 0.6980 | -0.1207 | 0.1804 |
| Age first birth 18- | -0.0618 | 0.0154 | -4.0100 | 0.0000 | -0.0921 | -0.0316 |
| Age first birth missing | -0.0299 | 0.0521 | -0.5700 | 0.5660 | -0.1321 | 0.0722 |
| Decision mother | -0.0504 | 0.0560 | -0.9000 | 0.3680 | -0.1600 | 0.0593 |
| Joint decision | -0.0208 | 0.0566 | -0.3700 | 0.7130 | -0.1318 | 0.0901 |
| Decision missing | -0.1066 | 0.0417 | -2.5600 | 0.0110 | -0.1882 | -0.0249 |
| Ethnicity regular | -0.0015 | 0.0693 | -0.0200 | 0.9830 | -0.1372 | 0.1343 |
| Ethnicity minority | -0.0295 | 0.0580 | -0.5100 | 0.6110 | -0.1432 | 0.0842 |
| Ethnicity missing | -0.0576 | 0.0686 | -0.8400 | 0.4020 | -0.1921 | 0.0769 |
| Catholic | 0.2024 | 0.0441 | 4.6000 | 0.0000 | 0.1161 | 0.2888 |
| Religion missing | 0.1859 | 0.0496 | 3.7500 | 0.0000 | 0.0887 | 0.2830 |
| Protestant | 0.0919 | 0.0456 | 2.0200 | 0.0440 | 0.0025 | 0.1813 |
| Christian | 0.1025 | 0.0364 | 2.8100 | 0.0050 | 0.0311 | 0.1739 |
| Muslim | 0.1409 | 0.0668 | 2.1100 | 0.0350 | 0.0100 | 0.2718 |
| Other | 0.0719 | 0.0572 | 1.2600 | 0.2080 | -0.0401 | 0.1840 |
| No religion | 0.0282 | 0.0673 | 0.4200 | 0.6760 | -0.1037 | 0.1600 |
| Delivery at home | -0.1947 | 0.0585 | -3.3300 | 0.0010 | -0.3095 | -0.0800 |
| Delivery missing | -0.2419 | 0.0566 | -4.2800 | 0.0000 | -0.3528 | -0.1311 |
| Delivery assistance missing | 0.1120 | 0.0597 | 1.8800 | 0.0610 | -0.0051 | 0.2291 |
| No delivery assistance | -0.0478 | 0.0766 | -0.6200 | 0.5330 | -0.1980 | 0.1024 |
| Skilled delivery assistance | 0.1503 | 0.0667 | 2.2500 | 0.0240 | 0.0196 | 0.2810 |
| Other delivery assistance | -0.0198 | 0.0415 | -0.4800 | 0.6340 | -0.1012 | 0.0616 |
| No prenatal care | -0.3602 | 0.0951 | -3.7900 | 0.0000 | -0.5466 | -0.1738 |
| Skilled prenatal care | -0.0174 | 0.0558 | -0.3100 | 0.7540 | -0.1268 | 0.0919 |
| Other prenatal care | -0.0724 | 0.0507 | -1.4300 | 0.1530 | -0.1718 | 0.0270 |
| Prenatal care missing | 0.0437 | 0.0114 | 3.8300 | 0.0000 | 0.0213 | 0.0660 |
| No postnatal check-up | -0.0755 | 0.0361 | -2.0900 | 0.0370 | -0.1463 | -0.0047 |
| Postnatal check-up missing | -0.1052 | 0.0620 | -1.7000 | 0.0900 | -0.2268 | 0.0163 |
| No vaccination | -0.2860 | 0.0717 | -3.9900 | 0.0000 | -0.4266 | -0.1454 |
| Vaccination missing | 0.0913 | 0.0325 | 2.8100 | 0.0050 | 0.0276 | 0.1549 |
| No vitamin A | -0.1398 | 0.0466 | -3.0000 | 0.0030 | -0.2311 | -0.0485 |
| Vitamin A missing | 0.0057 | 0.0776 | 0.0700 | 0.9420 | -0.1465 | 0.1578 |
| Health availability | 0.0933 | 0.0200 | 4.6600 | 0.0000 | 0.0541 | 0.1325 |
| Urbanization | 0.0918 | 0.0457 | 2.0100 | 0.0450 | 0.0022 | 0.1814 |
| GDP per capita | 0.0002 | 0.0001 | 3.2100 | 0.0010 | 0.0001 | 0.0004 |
| Centralized | -1.2633 | 0.3192 | -3.9600 | 0.0000 | -1.8890 | -0.6377 |
| Fee | -1.0964 | 0.3025 | -3.6200 | 0.0000 | -1.6894 | -0.5035 |
| Fee missing | -3.0744 | 0.2182 | -14.0900 | 0.0000 | -3.5020 | -2.6468 |
| Fertility rate | 0.4829 | 0.2338 | 2.0700 | 0.0390 | 0.0246 | 0.9412 |
| Number of conflicts | -0.0239 | 0.0104 | -2.3100 | 0.0210 | -0.0442 | -0.0036 |
| *Random intercepts* |  |  |  |  |  |  |
| National | 0.9565 | 0.2209 |  |  | 0.5235 | 1.3895 |
| Regional | 0.3828 | 0.0949 |  |  | 0.1968 | 0.5688 |
